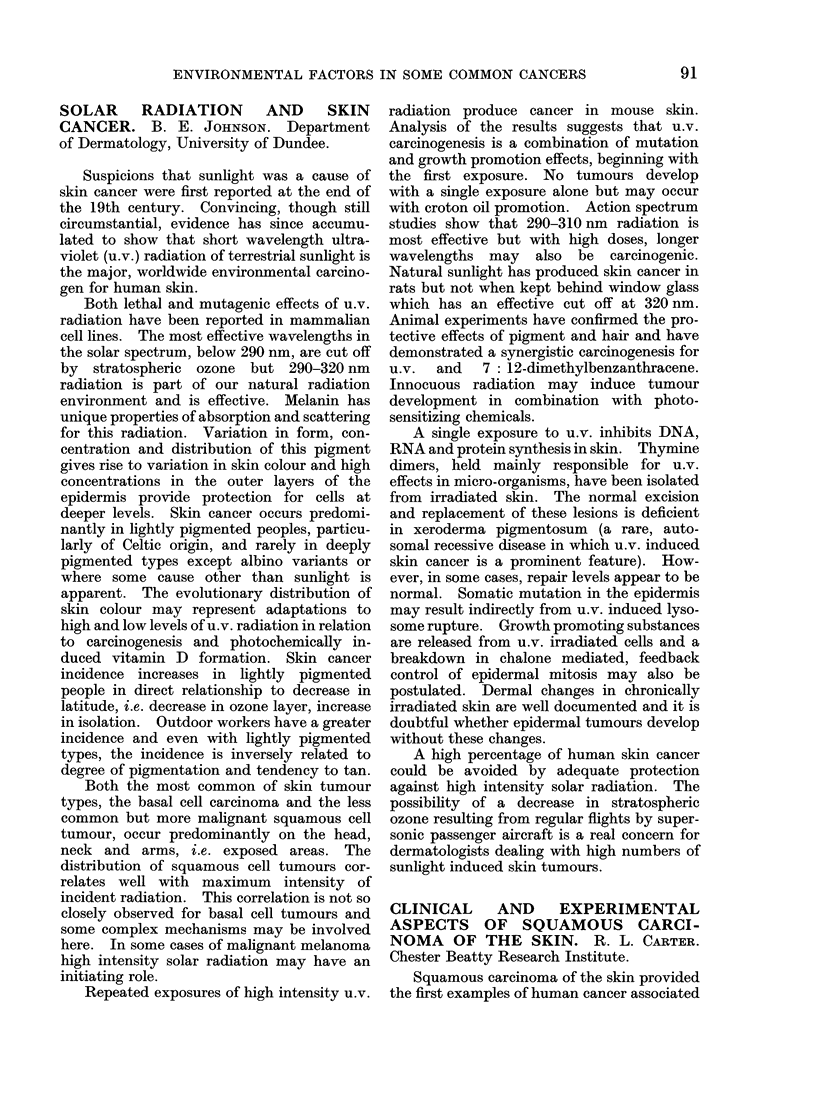# Solar radiation and skin cancer.

**DOI:** 10.1038/bjc.1973.118

**Published:** 1973-07

**Authors:** B. E. Johnson


**SOLAR RADIATION AND SKIN CANCER.** B. E. JOHNSON. Department of Dermatology, University of Dundee.

Suspicions that sunlight was a cause of skin cancer were first reported at the end of the 19th century. Convincing, though still circumstantial, evidence has since accumulated to show that short wavelength ultraviolet (u.v.) radiation of terrestrial sunlight is the major, worldwide environmental carcinogen for human skin.

Both lethal and mutagenic effects of u.v. radiation have been reported in mammalian cell lines. The most effective wavelengths in the solar spectrum, below 290 nm, are cut off by stratospheric ozone but 290–320 nm radiation is part of our natural radiation environment and is effective. Melanin has unique properties of absorption and scattering for this radiation. Variation in form, concentration and distribution of this pigment gives rise to variation in skin colour and high concentrations in the outer layers of the epidermis provide protection for cells at deeper levels. Skin cancer occurs predominantly in lightly pigmented peoples, particularly of Celtic origin, and rarely in deeply pigmented types except albino variants or where some cause other than sunlight is apparent. The evolutionary distribution of skin colour may represent adaptations to high and low levels of u.v. radiation in relation to carcinogenesis and photochemically induced vitamin D formation. Skin cancer incidence increases in lightly pigmented people in direct relationship to decrease in latitude, *i.e.* decrease in ozone layer, increase in isolation. Outdoor workers have a greater incidence and even with lightly pigmented types, the incidence is inversely related to degree of pigmentation and tendency to tan.

Both the most common of skin tumour types, the basal cell carcinoma and the less common but more malignant squamous cell tumour, occur predominantly on the head, neck and arms, *i.e.* exposed areas. The distribution of squamous cell tumours correlates well with maximum intensity of incident radiation. This correlation is not so closely observed for basal cell tumours and some complex mechanisms may be involved here. In some cases of malignant melanoma high intensity solar radiation may have an initiating role.

Repeated exposures of high intensity u.v. radiation produce cancer in mouse skin. Analysis of the results suggests that u.v. carcinogenesis is a combination of mutation and growth promotion effects, beginning with the first exposure. No tumours develop with a single exposure alone but may occur with croton oil promotion. Action spectrum studies show that 290–310 nm radiation is most effective but with high doses, longer wavelengths may also be carcinogenic. Natural sunlight has produced skin cancer in rats but not when kept behind window glass which has an effective cut off at 320 nm. Animal experiments have confirmed the protective effects of pigment and hair and have demonstrated a synergistic carcinogenesis for u.v. and 7 : 12-dimethylbenzanthracene. Innocuous radiation may induce tumour development in combination with photosensitizing chemicals.

A single exposure to u.v. inhibits DNA, RNA and protein synthesis in skin. Thymine dimers, held mainly responsible for u.v. effects in micro-organisms, have been isolated from irradiated skin. The normal excision and replacement of these lesions is deficient in xeroderma pigmentosum (a rare, autosomal recessive disease in which u.v. induced skin cancer is a prominent feature). However, in some cases, repair levels appear to be normal. Somatic mutation in the epidermis may result indirectly from u.v. induced lysosome rupture. Growth promoting substances are released from u.v. irradiated cells and a breakdown in chalone mediated, feedback control of epidermal mitosis may also be postulated. Dermal changes in chronically irradiated skin are well documented and it is doubtful whether epidermal tumours develop without these changes.

A high percentage of human skin cancer could be avoided by adequate protection against high intensity solar radiation. The possibility of a decrease in stratospheric ozone resulting from regular flights by supersonic passenger aircraft is a real concern for dermatologists dealing with high numbers of sunlight induced skin tumours.